# Chest X-ray versus chest computed tomography for outcome prediction in hospitalized patients with COVID-19

**DOI:** 10.1007/s11547-022-01456-x

**Published:** 2022-01-26

**Authors:** Andrea Borghesi, Salvatore Golemi, Alessandra Scrimieri, Costanza Maria Carlotta Nicosia, Angelo Zigliani, Davide Farina, Roberto Maroldi

**Affiliations:** Department of Medical and Surgical Specialties, Radiological Sciences and Public Health, University of Brescia, ASST Spedali Civili of Brescia, Piazzale Spedali Civili, 1, 25123 Brescia, Italy

**Keywords:** SARS-CoV-2, COVID-19, Chest X-ray, Computed tomography, Scoring system

## Abstract

The purpose of this study was to compare the prognostic value of chest X-ray (CXR) and chest computed tomography (CT) in a group of hospitalized patients with COVID-19. For this study, we retrospectively selected a cohort of 106 hospitalized patients with COVID-19 who underwent both CXR and chest CT at admission. For each patient, the pulmonary involvement was ranked by applying the *Brixia* score for CXR and the percentage of well-aerated lung (WAL) for CT. The *Brixia* score was assigned at admission (A-*Brixia* score) and during hospitalization. During hospitalization, only the highest score (H-*Brixia* score) was considered. At admission, the percentage of WAL (A-CT%WAL) was quantified using a dedicated software. On logistic regression analyses, H-*Brixia* score was the most effective radiological marker for predicting in-hospital mortality and invasive mechanical ventilation. Additionally, A-CT%WAL did not provide substantial advantages in the risk stratification of hospitalized patients with COVID-19 compared to A-*Brixia* score.

## Introduction

Ten months after the start of coronavirus disease (COVID-19) vaccination planning, the diffusion and virulence of the severe acute respiratory syndrome coronavirus 2 (SARS-CoV-2) are progressively decreasing in many parts of the world. On October 21, 2021, the number of new SARS-CoV2 infections in Italy was 3794, with 22 new admissions to intensive care units (ICUs), and 36 new deaths. There was a decrease in new infections by 90.7%, in new admissions to ICU by 63.3% for new admissions to ICUs, and in new deaths by 93.5% as compared to the peak of incidence observed on November 13, 2020 during the second wave of COVID-19 in Italy [1, 2].

Despite the progressive reduction in hospitalization and fatality rates in patients with COVID-19, ICU admissions and deaths are still observed [1]. Additionally, with the progressive lowering of temperatures in the coming months, an increase in new COVID-19 cases is anticipated.

Chest X-ray (CXR) and chest computed tomography (CT) are the most commonly used imaging techniques for the management (diagnosis, hospitalization, and follow-up) of patients with COVID-19 [3–5], and several authors have found that both modalities are useful predictors of patient outcome [6–12]. Recently, Sverzellati et al. [13] found that, in a simulated triage setting, the use of r-CXR (coronal image reconstructed from thin-section CT scan) in cases suspected of having COVID-19 was safe and helped optimizing both the use of radiology resources and patient management. However, to the best of our knowledge, the performance of CXR and chest CT for predicting adverse outcomes, such as invasive mechanical ventilation (IMV) and COVID-19 related mortality, has not yet been compared in the same cohort of hospitalized patients with COVID-19. Therefore, the aim of the present study was to retrospectively compare the prognostic value of CXR and chest CT at admission and during hospitalization in a group of patients with COVID-19.

## Materials and methods

To compare the prognostic value of CXR and chest CT, we selected a cohort of patients with COVID-19, as confirmed by real-time polymerase chain reaction (RT-PCR), admitted to our hospital during the second wave (from October, 2020 to February, 2021) and for whom information on adverse outcomes (using IMV and in-hospital mortality) was available. We enrolled only patients from the second wave of COVID-19 because during the first wave, we almost exclusively used CXR because of the high pre-test probability of the disease.

For the analysis, we selected only hospitalized patients who underwent both CXR and chest CT at admission (time interval between the two imaging modalities not exceeding 24 h). For each patient, CXR and chest CT performed at admission and CXRs performed during hospitalization were considered for the study. Each frontal chest projection was independently evaluated by a thoracic radiologist with 16 years of experience in thoracic imaging (A.B.), who ranked the pulmonary involvement of the disease based on a dedicated 18-point severity scale (the *Brixia* score) previously described by Borghesi and Maroldi [6]. On chest CT, the well-aerated lung (WAL) was quantified using a dedicated three-dimensional software (Syngo CT Pulmo 3D, Siemens Healthcare GMBH) by applying a method similar to that previously described by Colombi et al. [11]. The software-based calculation of the CT percentage of WAL at admission (A-CT%WAL) was independently performed by a radiology resident (S.G.) with three years of experience in thoracic imaging and one year of experience in using the software.

The data are presented as numbers (%) or as mean ± standard deviation for normally distributed data or as median and interquartile range (IQR) for non-normally distributed data. Relationships between adverse outcomes (using IMV and in-hospital mortality) and A-*Brixia* score (the score assigned on CXR at admission), H-*Brixia* score (the highest score assigned on CXRs during hospitalization), and A-CT%WAL were tested using univariate and multivariable logistic regression analyses. The predictive power of A-*Brixia* score, H-*Brixia* score, and A-CT%WAL was expressed as the area under the curve (AUC). Statistical analysis was conducted using MedCalc® Statistical Software version 20.009 (MedCalc Software Ltd, Ostend, Belgium). Statistical significance was set at p values < 0.05.

## Results

**Fig. 1:**
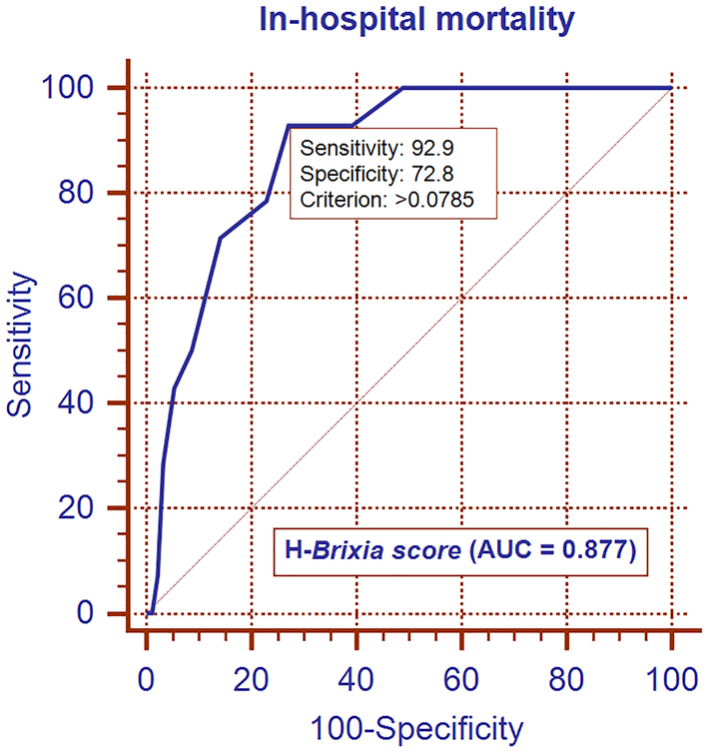
ROC curve for in-hospital mortality: model based on H-*Brixia score*

**Fig. 2:**
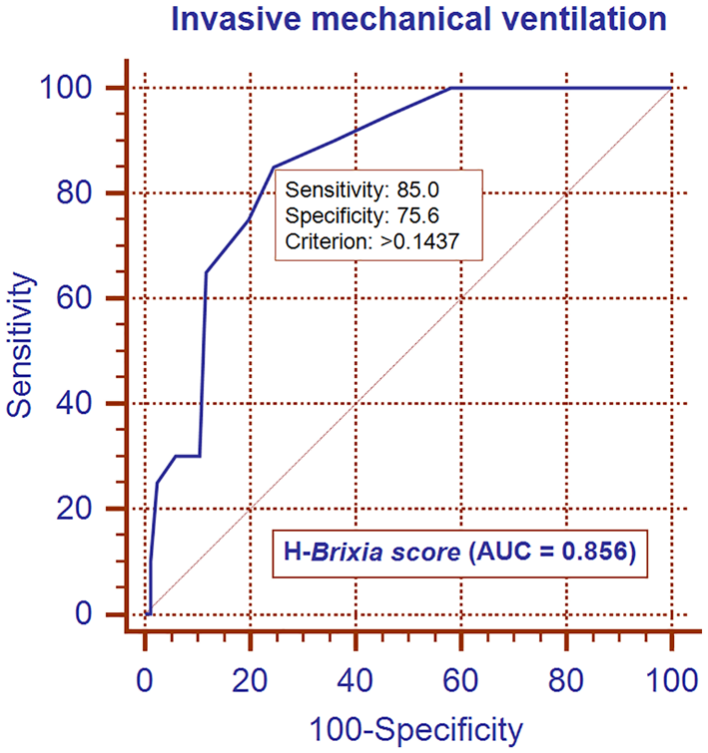
ROC curve for invasive mechanical ventilation: model based on H-*Brixia score*

According to the study inclusion criteria, we enrolled 106 consecutive patients (76 men and 30 women) with a mean age of 67.5 ± 13.8 years. Of the included patients, 14 (13.2%) died and 20 (18.9%) required IMV. The median values of the A-*Brixia* score, H-*Brixia* score, and A-CT%WAL were 4 (IQR, 2–4), 7 (IQR, 4–10), and 74.5% (IQR, 56–84%), respectively. The relationships between adverse outcomes and the A-*Brixia* score, H-*Brixia* score, and A-CT%WAL on logistic regression analyses are listed in Tables 1 and 2. The H-*Brixia* score was the most effective radiological marker for predicting in-hospital mortality (AUC, 0.877) and IMV (AUC, 0.856). Particularly, in multivariable analysis (using a stepwise approach), only the H-*Brixia* score was an independent predictive marker for adverse outcomes (Tables 1 and 2). Based on receiver operating characteristic curves, the optimal cutoff value for the H-*Brixia* score was 8 points with a sensitivity for in-hospital mortality and IMV of 92.9% and 85.0%, respectively (Figs. 1 and 2). Additionally, using the DeLong test, the AUC of A-*Brixia* score and A-CT%-WAL for predicting adverse outcomes did not show a significant difference (p > 0.415).

**Table 1:** Univariate and multivariable regression analyses for in-hospital mortality

Radiological marker	Univariate analysis				Multivariable analysis			
	coefficient	*P* value	OR	AUC	coefficient	*P* value	OR	AUC
A-*Brixia score*	0.239	0.001	1.270	0.721	–	–	–	–
H- *Brixia score*	0.457	< 0.001	1.579	0.877	0.457	< 0.001	1.579	0.877
A-CT%WAL	− 0.060	0.001	0.942	0.759	–	–	–	

*OR* odds ratio, *AUC* area under the curve

**Table 2:** Univariate and multivariable regression analyses for invasive mechanical ventilation

Radiological marker	Univariate analysis				Multivariable analysis			
	coefficient	*P* value	OR	AUC	coefficient	*P* value	OR	AUC
A-*Brixia score*	0.244	< 0.001	1.276	0.736	–	–	–	–
H- *Brixia score*	0.415	< 0.001	1.515	0.856	0.415	< 0.001	1.515	0.856
A-CT%WAL	− 0.042	0.005	0.959	0.704	–	–	–	

*OR* odds ratio, *AUC* area under the curve

## Discussion

Chest imaging modalities, specifically CXR and chest CT, play a key role in the management of patients with COVID-19. In February 2021, the World Health Organization (WHO) published a simple guide on the use of chest imaging techniques in patients with confirmed or suspected COVID-19[3]. For symptomatic patients with COVID-19, the WHO recommends chest imaging in addition to clinical and laboratory examinations to decide between patient discharge and hospitalization or to identify patients requiring specific therapeutic management [3].

However, one of the most challenging questions about chest imaging in symptomatic patients with COVID-19 is which imaging modalities between CXR and chest CT are the most effective for improving risk stratification of infected patients and predicting disease progression. Until now, the answer to this question remains unresolved as the published data on chest imaging lack a comparative analysis of the prognostic value of CXR and chest CT.

Although it is well known that chest CT is the most sensitive imaging technique for the detection of lung abnormalities, and quantitative CT analysis provides useful information for predicting disease progression [11, 12], CXR has several advantages for the management of hospitalized patients with COVID-19 [5, 6, 14]. The main advantage is the possibility of using CXR as a diagnostic tool to monitor (“day by day”) the course of the disease, especially in the most critically affected patients [5, 6, 14].

In our study cohort of hospitalized patients with COVID-19, we found that in the multivariable analysis, the H-*Brixia* score (the highest score assigned on CXRs during hospitalization) was the only independent predictor of adverse outcomes in hospitalized patients with COVID-19. In particular, the H-*Brixia* score exhibited excellent power in predicting in-hospital mortality and the need for IMV (Figs. 1 and 2). We also found that, at admission, chest CT with the quantitative assessment of the extent of WAL (A-CT%WAL) did not provide substantial advantages in the risk stratification of COVID-19 patients compared to CXR with semiquantitative assessment of the disease severity (A-*Brixia* score) (Tables 1 and 2). Therefore, similar to the study of Sverzellati et al. [13], we can state that in symptomatic patients with COVID-19 (confirmed by RT-PCR), chest CT should not be considered the first-line imaging modality to evaluate the extent of pulmonary involvement and decide between discharge and hospitalization because its prognostic power does not differ substantially from that of CXR.

Obviously, chest CT remains the most suitable radiological modality for confirming or excluding COVID-19 pneumonia, specifically in symptomatic patients with clinical suspicion of COVID-19 not confirmed by RT-PCR [3, 4, 15, 16]. In addition, chest CT remains the method of choice for confirming or excluding thoracic complications and sequelae of COVID-19, such as pulmonary embolism and fibrosis [17, 18].

The main limitations of our study include the retrospective design of the analysis and the relatively small sample size (106 patients). Another limitation is that the prognostic value of chest CT was evaluated only at admission because chest CT was performed in a limited number of patients during hospitalization, specifically when a pulmonary embolism was suspected.

In conclusion, this study confirmed that the H-*Brixia* score is an excellent marker for predicting adverse outcomes in hospitalized patients with COVID-19. Additionally, this study showed that at admission, the prognostic value of chest CT is not superior to that of CXR. These results are of great importance and will help both radiologists and clinicians in choosing the appropriate imaging modalities for management of symptomatic patients with COVID-19.
